# Animal models for type 1 and type 2 diabetes: advantages and limitations

**DOI:** 10.3389/fendo.2024.1359685

**Published:** 2024-02-20

**Authors:** Raj Singh, Mazaher Gholipourmalekabadi, Sasha H. Shafikhani

**Affiliations:** ^1^ Department of Medicine, Division of Hematology, Oncology, & Cell Therapy, Rush University Medical Center, Chicago, IL, United States; ^2^ Cellular and Molecular Research Center, Iran University of Medical Sciences, Tehran, Iran; ^3^ Department of Medical Biotechnology, Faculty of Allied Medicine, Iran University of Medical Sciences, Tehran, Iran; ^4^ Cancer Center, Rush University Medical Center, Chicago, IL, United States

**Keywords:** diabetes mellitus, diabetes, type 1 diabetes (T1D), type 2 diabetes (T2D), animal models, animal models for type 1 diabetes (T1D), animal models for type 2 diabetes (T2D)

## Abstract

Diabetes mellitus, commonly referred to as diabetes, is a group of metabolic disorders characterized by chronic elevation in blood glucose levels, resulting from inadequate insulin production, defective cellular response to extracellular insulin, and/or impaired glucose metabolism. The two main types that account for most diabetics are type 1 diabetes mellitus (T1DM) and type 2 diabetes mellitus (T2DM), each with their own pathophysiological features. T1D is an autoimmune condition where the body’s immune system attacks and destroys the insulin-producing beta cells in the pancreas. This leads to lack of insulin, a vital hormone for regulating blood sugar levels and cellular glucose uptake. As a result, those with T1D depend on lifelong insulin therapy to control their blood glucose level. In contrast, T2DM is characterized by insulin resistance, where the body’s cells do not respond effectively to insulin, coupled with a relative insulin deficiency. This form of diabetes is often associated with obesity, sedentary lifestyle, and/or genetic factors, and it is managed with lifestyle changes and oral medications. Animal models play a crucial role in diabetes research. However, given the distinct differences between T1DM and T2DM, it is imperative for researchers to employ specific animal models tailored to each condition for a better understanding of the impaired mechanisms underlying each condition, and for assessing the efficacy of new therapeutics. In this review, we discuss the distinct animal models used in type 1 and type 2 diabetes mellitus research and discuss their strengths and limitations.

## 1 Introduction

Diabetes mellitus, commonly referred to as diabetes, is a group of metabolic disorders characterized by chronic elevation in blood glucose levels, resulting from inadequate insulin production, impaired cellular response to extracellular insulin, and/or impaired glucose metabolism (1–3). Nearly 10% of the world’s population is impacted by diabetes. As of 2021, about 537 million adults globally are living with this condition, a figure expected to rise to 643 million by 2030 and 783 million by 2045, based on data from the International Diabetes Federation (accessed on January 29, 2024).

Diabetes has been associated with severe and potentially life-threatening complications, such as cardiovascular diseases, neuropathy, retinopathy, nephropathy, hearing impairment, and chronic ulcers which can lead to lower extremity amputation with high mortality rates (1, 4, 5). In addition, diabetes has also been associated with increased risk of certain cancers and is a recognized risk factor for mental disorders, such as Alzheimer’s disease (6, 7). Moreover, diabetes significantly increases the susceptibility to bacterial, fungal, and viral infections, which in turn, contribute significantly to the morbidity and mortality rates among individuals with diabetes (8–13).

Diabetes is a complex disease that can take multiple different forms. The two main types that account for most diabetics are type 1 diabetes mellitus (T1DM) and type 2 diabetes mellitus (T2DM), which account for approximately 9.5% and 90% of all diabetes respectively (1, 14).

T1DM, (a.k.a., juvenile diabetes or insulin-dependent diabetes), is an autoimmune disorder whereby the body views its insulin-producing pancreatic beta (β) cells as a foreign threat, and subsequently, directs the immune system to attack and destroy them, leading to insulin insufficiency (15, 16). The exact underlying cause(s) remain uncertain, but T1DM typically manifests during childhood or early adolescence (14). Patients diagnosed with T1DM must rely on exogenous insulin administration to allow glucose cellular uptake cells and metabolism (14, 16).

T2DM is not an autoimmune condition. Rather, it is a metabolic disorder that develops through a combination of lifestyle and genetic factors (1). Other than the characteristic high blood glucose levels, it is defined by insulin resistance where cells do not respond appropriately to insulin, or relative insulin deficiency where the body does not produce enough insulin (15, 16). There are several risk factors that can increase the likelihood of T2DM. These factors include ageing, sedentary lifestyle, obesity, metabolic syndrome and its components, ethnicity, depression, high blood pressure, family history, and low socioeconomic status (17, 18). Proper lifestyle modifications, including consuming a balanced nutritional diet, losing weight, and engaging in routine exercise, can play a crucial role in the management of T2DM and may even allow some individuals to control diabetes without the need for medications (19, 20). This approach is often referred to as “lifestyle management” or “lifestyle therapy” for diabetes (19, 20).

With this alarming increase in diabetes cases, ongoing research into its causes and innovative treatments remains crucial. Animal models have and continue to be important tools for discovering defective mechanisms underlying co-morbidities associated with both type 1 and type 2 diabetes in patients and for evaluating novel therapeutics to treat these diseases (21, 22). However, every animal model has its own unique advantages and limitations; therefore, selecting an appropriate model for addressing a specific problem or situation can vastly influence the study results and interpretation. With the number of animal models rising, it is critical that we continue to consider their potential roles within the numerous aspects of diabetes research and discovery.

Considering the distinct differences between T1DM and T2DM, it is of imperative for researchers to utilize tailored animal models specific to each condition. This approach is essential to gain a deeper understanding of the underlying mechanisms and to assess the effectiveness of therapeutic interventions designed for these particular types of diabetes. In this review article, we categorize diabetes animal models based on the methodologies used for their development. These methodologies include chemically induced, genetically/spontaneously induced, virus-induced, surgically constructed, and diet/nutrition-induced animal models. We delve into these distinct animal model categories used in research for type 1 and type 2 diabetes mellitus and discuss their respective strengths and limitations.

## 2 Type 1 diabetic animal models

In the T1DM research, major animal models include those generated chemically, genetically or by spontaneous mutations, surgically, and by employing viral infections. These animal models are discussed below and summarized in **Tables 1**–**4**.

**Table 1:** Chemically Induced T1DM Animal Models.

Animal	Chemical	Advantages	Limitations
**Rat**	Alloxan	Cost-effective (23) Ease of handling (23) Shares similar numerous pathophysiology and pathological features with humans (23) A reduced amount of stress by human contact (23)	Loose skin with dense hair (23, 24) Healing of wound occur via wound contraction (23, 24) Partial-thickness wounds are difficult to create because skin is too thin (23, 24) Chemical can be toxic at other organs of the body (25) Hyperglycemia mainly develops through the direct cytotoxic action on pancreatic β-cells and insulin deficiency rather than the adverse effects of insulin resistance (24)

	STZ* Cyclophosphamide MLD-STZ*		
**Mouse**	Alloxan STZ Cyclophosphamide MLD-STZ	Cost-effective (23) Easy housing (23) Shares similar numerous pathophysiology and pathological features with humans (23) Less rate of morbidity (23)	Loose skin with dense hair (23, 24) Wound healing occurs primarily by wound contraction (23, 24) Partial-thickness wounds are difficult to create because skin is too thin (23, 24) Chemical can be toxic at other organs of the body (25) Hyperglycemia mainly develops through the direct cytotoxic action on pancreatic β-cells and insulin deficiency rather than the adverse effects of insulin resistance (24, 26)
**Rabbit**	Alloxan STZ Cyclophosphamide MLD-STZ	More cost effective than other larger animals (23, 24) Share similar metabolic and pathological profile of burn wounds to that of humans (23, 24)	Difficult to produce irreversible diabetes (27) Risk of infections and morbidity compared to mouse and rat (23, 24)
**Dog**	Alloxan STZ MLD-STZ	Shares similar numerous pathophysiology and pathological features with humans (23) Mimics similar symptoms and pathophysiology of thermal burn wounds (23, 24)	Expensive animal model (23) Loose skin (23) Difficult to produce irreversible diabetes (27) May develop moderate hyperglycemia without clear alterations in body weight or blood insulin levels (26)
**Pig**	Alloxan STZ MLD-STZ	Anatomy and physiology of diabetes matches closely to that of humans (27) Useful for studying T1DM* and T2DM* (27)	Expensive animal model with more cost for care and housing (23) Administration of anesthesia requires a skilled veterinarian (23) Requires higher doses to produce diabetic conditions (27) Difficult to produce irreversible diabetes (27)
**Primate**	Alloxan STZ MLD-STZ	A multiple low-dose STZ model has been described in primates and shown beneficiary results for T1DM (27) Shares similar numerous pathophysiology and pathological features with humans (27)	Expensive animal model (27) Difficult to produce irreversible diabetes (27) Administration of a drug requires a skilled handler and researcher (23)

*STZ, Streptozotocin; T1DM, Type 1 Diabetes Mellitus; T2DM, Type 2 Diabetes Mellitus; MLD-STZ, Multiple Low Dose- Streptozotocin.

**Table 2:** Genetically/spontaneously induced T1D animal models.

Animal	Gene(s)	Advantages	Limitations
NOD* Mouse	Polygenic model that displays hyperglycemia, leukocytic infiltration, and destruction of the pancreatic β-cells (27)	Widely used to study T1DM* (28) Exploring genetics of T1DM (27)	Expensive (27, 29) Difficult to handle (27, 29)
KDP* Rat	Formed from a nonsense mutation in the Cbl-b (30)	Rapid onset of diabetes with no significant T-lymphopenia (31) Both male and female rats develop T1DM at high and similar frequencies of approximately 70% (26)	Expensive (27, 31) Only a handful of studies have been based on this model, which were largely genotype studies (31)
LETL* Rat	Mirrors the pathology and phenotypic nature of human T1DM such as the sudden onset of polyuria, polyphagia, and hyperglycemia (32)	First discovered rat model that spontaneously formed an autoimmune destruction of its islet β-cells (26) Spontaneously forms autoimmune diabetes without lymphopenia (33)	Developing rapid diabetes at only a 20 percent rate (26) Expensive (32, 33) Does not completely replicate the T1DM conditions seen in human subjects (32, 33)
**LEW-IDDM Rat**	Spontaneously develops insulin-dependent autoimmune diabetes through pancreatic β cell apoptosis (34)	Can create T1DM in male and females with indistinguishable frequency (35) Used to investigate possible treatments that may prevent the destruction of pancreatic β-cells (27)	Expensive (27, 34) May not completely mimic the T1DM condition seen in humans (27, 34, 36, 37)
BB* Rat	Presents with hyperglycemia and ketoacidosis, which is associated with the clinical onset of T1DM (27)	BB rat is preferably utilized for investigating islet transplantation tolerance induction (34, 38) Animal models show hypoinsulinemia, ketonuria, and weight loss (34)	Diabetes is associated with T-cell lymphopenia (26) Expensive (26)
**Akita Mouse**	Develops diabetes due to a mutation affecting insulin 2. Exhibits key characteristics of T1DM in human, such as insulin deficiency and hyperglycemia due to b cell destruction (39, 40). Commonly used to investigate diabetic related complications such as modest levels of albuminuria and mild-to-moderate glomerular mesangial expansion (41)	Genetic clarity: Caused by a single mutation in insulin 2. This can simplify the study of diabetes pathogenesis compared to polygenic models (42)	Not due to an autoimmune process which occurs in human. Has a greater capacity for β cell regeneration compared to humans, which may affect the translation of findings from Akita mice to human T1DM (43)

*BB, Bio-Breeder; KDP, Komeda Diabetes-Prone; LETL, Long Evans Tokushima Lean; LEW-IDDM, LEW.1AR1-iddm; NOD, Nonobese Diabetic; T1DM, Type 1 Diabetes Mellitus.

**Table 3:** Virally induced T1DM animal models.

Animal	Virus Type	Advantage	Limitations
**Mouse**	EMC* virus Coxsackie virus LCMV*	Clinical diabetes formed from the destruction of pancreatic β-cells after EMC viral induction (44) Coxsackie B virus is passed in pancreatic β-cells before infection develops (45) Develops T1DM* (44)	Complete mechanism for getting diabetes is unknown (45) Lack of specificity and may affect other organs (33, 44–46) Ethical considerations for safety to prevent accidental infections or outbreaks (33)
**Hamster**	EMC virus Coxsackie virus LCMV*	Clinical diabetes formed from the destruction of pancreatic β-cells after EMC viral induction (45) Coxsackie B virus is passed in pancreatic β-cells before infection develops (45) LCMV induces immune mediated destruction causing T1DM (33, 34)	Complete mechanism for getting diabetes is unknown (45) Lack of specificity and may affect other organs (33, 44–46) Ethical considerations for safety to prevent accidental infections or outbreaks (33)
**Monkey**	EMC virus Coxsackie virus	Clinical diabetes formed from the destruction of pancreatic β-cells after EMC viral induction (45) Coxsackie B virus is passed in pancreatic β-cells before infection develops (45)	Complete mechanism for getting diabetes is unknown (45) Lack of specificity and may affect other organs (33, 44–46) Ethical considerations for safety to prevent accidental infections or outbreaks (33)
**Rat**	RCMV* KRV*	RCMV model utilized to study the mechanisms by which viral infections can induce or worsen T1DM (33, 34, 47) KRV model is useful for investigating the possible connection between viral infections and the formation of type 1 diabetes mellitus (33, 34)	Complete mechanism for getting diabetes is unknown (45) Lack of specificity and may affect other organs (33, 44–46) Ethical considerations for safety to prevent accidental infections or outbreaks (33)

*EMC, Encephalomyocarditis; T1DM, Type 1 Diabetes Mellitus; LCMV, Lymphocytic Choriomeningitis Virus; RCMV, Rat Cytomegalovirus; KRV, Kilham Rat Virus.

**Table 4:** Surgical T1D anima models.

Animal	Surgery Type	Advantage	Limitations
**Rat**	Pancreatomy Islet Transplantation Thymectomy	Reflects the isolated effects of a reduced β cell mass (48) Cost effective, easy handling, and housing (49)	Partial-thickness wounds are difficult to create because skin is too thin (23, 50) High possibly of exocrine acinar cells being excised too (48)
**Mouse**	Pancreatomy Islet Transplantation Thymectomy	Reflects the isolated effects of a reduced β cell mass (48) Cost effective and easy housing (49)	Partial-thickness wounds are difficult to create because skin is too thin (23, 50) High possibly of exocrine acinar cells being excised as well (48)
**Rabbit**	Pancreatomy Islet Transplantation Thymectomy	Easy to handle and house (51) May work with larger sample of animals (51)	Live in an artificial environment (51) Have a standardized diet (51) High cost (49) High technical skills needed (49)
**Dog**	Pancreatomy Islet Transplantation Thymectomy	Model is a reliable method to induce hyperglycemia (27) Large size and longer lifespan (27)	Invasive surgery (27) Increased chance of hypoglycemia (27) High cost (49) High technical skills needed (49)
**Pig**	Pancreatomy Islet Transplantation Thymectomy	Pancreatectomy followed by autotransplantation of the isolated islets largely matches that seen in human diabetics (27)	Invasive surgery (23, 27) Increased chance of hypoglycemia (23, 27) High cost (49) High technical skills needed (49)
**Primate**	Pancreatomy Islet Transplantation Thymectomy	Higher lifespans (48, 52) Remarkable similarities to the human physiology (52)	Limited availability (52) Ethical concerns (48) High cost for handling and housing (52) High technical skills needed (49)

*T1DM, Type 1 Diabetes Mellitus.

### 2.1 Chemically induced T1D animal models

There are several chemically induced animal models for T1DM. The most common chemicals used to generate T1DM animals are alloxan and streptozotocin (STZ), but other chemicals such as cyclophosphamide and multiple low-dose streptozotocin (MLD-STZ) have also been. These animal models are discussed below and summarized in **Table 1**.

Streptozotocin and Alloxan are the most frequently used agents to chemically induce T1DM in animals (53). Both diabetogenic agents are toxic to pancreatic islet β cells which can consequently induce hyperglycemia within a few days (22). The dose required for inducing diabetes by these drugs depends on the animal species, route of administration, and nutritional status (53).

Streptozotocin (STZ) is a nitrosourea related antibiotic and antineoplastic drug that is produced by *Streptomyces achromogenes* (26). It destroys insulin-producing pancreatic β cells by DNA fragmentation through alkylation and alteration of macromolecules; thereby, causing insulin-dependent like diabetic conditions like hypoinsulinemia and hyperglycemia (54, 55). STZ may prevent DNA synthesis in both mammalian and bacterial cells by causing DNA fragmentation (56). This drug induces diabetes in animal models gradually within a few days, and it is the most common chemical used for the induction of diabetes in murine (particularly rats) and large animals (26, 57–62) (**Table 1**). This cytotoxic death of pancreatic β cells can be explained based on DNA methylation through the formation of carbonium ion, which ultimately results in NAD+ depletion, nitric oxide production, and free radical formation (55). The STZ chemically induced rodent model reproduces many of the acute and chronic complications of diabetes in humans (22). However, STZ also has some limitations that need to be considered. Due to its strong oncogenic actions, precautions must be taken to avoid danger to the investigators (49, 56). In addition has shown to cause insulinoma and elevated incidence of kidney and liver tumors in some experiments (49, 56).

Alloxan is a urea derivative that induces selective necrosis within pancreatic islet β-cells (49). Alloxan generates its toxic effects by creating highly reactive hydroxyl radicals that can cause DNA fragmentation (63). Alloxan also mediates the selective inhibition of glucose-stimulated insulin secretion and decreases hepatic glycogen storage within a few days after administration (25, 64). It is used to produce experimental diabetes in various animals such as rabbits, rats, mice, pigs, and dogs (27) (**Table 1**). The advantages of alloxan is its cost-effectiveness and its efficacy in creating animal models that mimic the human condition of T1DM (23). In addition, the hyperglycemia primarily results through the cytotoxicity of pancreatic islet β cells rather than insulin resistance, which is what is normally observed in type 1 diabetic patients (24). However, researchers must have sufficient training in managing alloxan-induced diabetic animals and should be cognizant of the potential health hazards posed to the animals by this chemical. The survival rates of alloxan induced T1DM animals remains a key challenge, due to the risk of lethal hypoglycemia triggered by alloxan injection (65). However, because STZ is more stable in solution, effective in more species, and offers greater reproducibility, it is the preferred drug of choice over alloxan to cause T1DM in animals (55).

Cyclophosphamide and multiple low-dose streptozotocin (MLD-STZ) have also been employed to generate T1DM animal models. Cyclophosphamide, an alkylating compound, directly damages pancreatic β cells through its DNA cross-linking actions and also triggers autoimmune reactions against pancreatic β cells, thus mimicking T1DM condition in human (22, 56). Cyclophosphamide-induced T1DM animals allow researchers to explore the immune mechanisms associated with T1DM. Moreover, the MLD-STZ approach - where small doses of STZ are given to animals (particularly mice) over multiple days - offers a more controlled way to induce T1DM in rodents (22, 24, 26). The MLD-STZ model closely resembles the gradual pancreatic β cell destruction seen in human T1DM patients (22, 24, 26).

There are also limitations with all these chemically induced animal models that investigators need to consider. These limitations include lack of disease heterogeneity, species differences, standardization challenges of achieving consistency and reproducibility, narrow focus on specific pathways, and ethical considerations (22, 26).

### 2.2 Genetically/spontaneously induced T1DM animal models

Animal models for T1DM have been developed through both spontaneous induction and genetic engineering. Some examples of these animal models include the Long Evans Tokushima Lean (LETL) rat, the Nonobese diabetic (NOD) mouse, Komeda diabetes-prone (KDP) rat, LEW.1AR1-iddm (LEW-IDDM) rat, and the Bio-Breeder (BB) rat (26, 34). These animal models are discussed below and summarized in **Table 2**.

The LETL (Long Evans Tokushima Lean) rat was the first rat model to spontaneously undergo autoimmune destruction of its islet β cells, leading to the development of diabetes at a frequency of 20 percent (26). Initial genetic analysis identified at least two recessive genes responsible for the induction of insulitis, one of which is closely linked with rat MHC RT1^u^ haplotype (32). LETL is widely used for investigating T1DM because it closely mirrors the pathology and phenotypic characteristics of human type 1 diabetics, such as sudden onset of polyuria, polyphagia, and hyperglycemia (32). In addition, this model spontaneously generates autoimmune diabetes without T cell lymphopenia, which is rarely seen in type 1 diabetic patients (33). While the LETL rat model offers valuable insights, there are notable constraints, including the low frequency of diabetes development in the rat, the substantial cost associated with these animals, and their inability to fully mimic human Type 1 diabetic mellitus conditions (32, 33). For instance, the LETL rat naturally exhibits autoimmune destruction of β-cells, but the pace and the pathophysiological mechanisms may not align with human experiences (32, 33). Furthermore, the LETL rats’ response to potential treatments or therapies may not always parallel human reactions, complicating direct comparisons (36).

The NOD mouse model is a polygenic model of T1D, which mimics many genetic and immunological traits seen in T1D human patients, such as hyperglycemia and immune cell-driven destruction of pancreatic β cells (27, 28). This model is one of the most widely used animal models for studying T1D, and it is particularly useful for understanding the genetics of T1DM (26, 27). However, there are also limitations with this animal model that should be considered, such as, its high costs, handling difficulties, and high vulnerability of these animals to infection (27, 29).

The KDP rat is another valuable animal model frequently used in T1D. It displays remarkable resemblance to human T1D, especially with regard to immune-mediated destruction of pancreatic β cells and its rapid onset of diabetes without marked T cell lymphopenia (31). The origin of this spontaneous model is a mutation in the Casitas B-lineage lymphoma proto-oncogene-b (Cbl-b) (30). Both genders of KDP rats develop type 1 diabetes at high frequency, with approximately 70 percent manifesting the disease (26). Despite its promise, the high cost of these animals has limited their use to only a handful of largely genotype focused research studies (31).

The LEW.1AR1-iddm (a.k.a., LEW-IDDM) rat arose following a spontaneous mutation associated with the telomeric region of rat chromosome 1 (*Iddm8*) in a colony of LEWIS.1AR1 rats (35, 66). LEW-IDDM develops insulin-dependent autoimmune diabetes through pancreatic β cell apoptosis (34). Both male and female rats develop type 1 diabetes with nearly identical frequencies, and this unique characteristic differentiates this model from other spontaneously induced animal models used for T1DM (35). This model is useful for early prediction of T1DM, focusing on disease prevention rather than cure (37). This rat model also allows researchers to investigate possible treatments that may prevent the destruction of β cells (27). Unlike other models, this group does not show autoantibodies against glutamic acid decarboxylase (GAD) or IA-2 at increased rates, and instead, it displays infiltrative immune cells attacking only the pancreas and no other glands (36). Like most other spontaneously autoimmune animal models, this model is expensive and may not completely mimic the type 1 diabetic condition seen in human diabetics (27, 34, 36, 37).

The Bio-Breeder (BB) rat model spontaneously develops diabetes with a high frequency between 60 to 120 days of age, displaying hyperglycemia and ketoacidosis, which are commonly seen at the onset of T1DM (27). This model also exhibits hypoinsulinemia, ketonuria, and weight loss (34). Researchers often employ the BB rat to study the induction of islet transplantation tolerance (34, 38). Despite these obvious advantages, the model has its drawbacks, including its high cost, and the diabetes in these rats is associated with T-cell lymphopenia, which is uncommon in type 1 diabetes in humans. Specifically, the rats lack CD8+ T-cells and have significantly reduced CD4+ T-cells, a condition not usually seen in humans (26).

The Akita mouse is a spontaneous model for T1DM, specifically carrying a mutation in the Ins2 gene that leads to misfolded insulin and subsequent pancreatic β cells death (39, 40). This model exhibits some of the key characteristics of human T1D, such as insulin deficiency and hyperglycemia. This model is commonly used to investigate diabetic related complications such as modest levels of albuminuria and mild-to-moderate glomerular mesangial expansion (41).

The Akita mouse model is beneficial for studying T1DM because genetic factors may influence susceptibility to diabetic nephropathy like in human disease (41). In addition, genetic clarity, due to a single mutation in this model, is advantageous, as it offers a clear genetic basis for the diabetes phenotype which can simplify the study of diabetes pathogenesis compared to polygenic models (42). In addition, the development of diabetes in the Akita mouse does not require chemical induction or other external interventions, which mirrors the spontaneous nature of human T1DM. This animal model also has its own limitations. For example, unlike human T1DM, which is primarily an autoimmune disease, the diabetes in the Akita mouse is not due to an autoimmune process. This limits its use for studying autoimmune aspects of T1DM. In addition, Akita mice have a greater capacity for β cell regeneration compared to humans, which may affect the translation of findings from Akita mice to human T1DM (43).

### 2.3 Virally induced T1D animal models

Viruses have also been used as a tool to target and destroy β cells in the pancreas, in order to generate T1D animal models. Notable viruses used in these animal models include the Coxsackie B virus (CBV), Encephalomyocarditis virus (EMCV), Lymphocytic choriomeningitis virus (LCMV), Cytomegalovirus (RCMV), and the Kilham virus (34, 46, 47). These animal models are described below and summarized in **Table 3**.

The CBV was originally discovered to be the cause of diabetic ketoacidosis in a healthy nondiabetic 10-year old child, and was shown to induce T1D in mice by causing necrosis in pancreatic β cells (47). This virus is the most predominantly found enterovirus in the diabetic individuals (46, 67). The mechanism by which CBV induces T1D in mice involves the release of a sequestered islet antigen resembling the autoantigen glutamic acid decarboxylase in human, which reactivates resting autoreactive T cells, thereby enhancing the autoimmune assault on pancreatic β cells (44). Additionally, CBV is known to upregulate the pancreatic expression of glutamic acid decarboxylase 65, a significant target in the autoimmune response of both humans and NOD mice (34, 45, 47).

EMCV is a picornavirus which has been shown to infect and cause necrosis in pancreatic β cells, inducing insulin-dependent hyperglycemia in mice and hamsters (34, 68).

LCMV, an arenavirus, can produce persistent infections in mice and infect pancreatic β cells by inducing immune mediated destruction causing T1DM (33, 34). Researchers use the LCMV induced animal model to study the diabetes pathogenesis and the immune response to viral infections (34, 45, 47).

RCMV, a herpesvirus, can initiate diabetes in susceptible stains of rats by involving immune-mediated pancreatic β cell destruction. Researchers use this model to study the mechanisms by which viral infections can induce or exacerbate T1DM (34, 45, 47).

KRV is a natural virus that can infect certain rat strains and induce diabetes through the inflammation of pancreatic β cells. Like other viral models, the KRV model is valuable for investigating the possible connection between viral infections and the formation of type 1 diabetes mellitus (33, 34, 45).

The use of viral models in the investigation of T1DM does come with certain limitations that warrant careful consideration. For example, the correlation between viruses and the development of diabetes in humans is complex and not as definitively understood as it might be in animal studies, potentially reducing the direct applicability of these findings to human conditions (33, 34). Moreover, viruses used in these models also have systemic effects, leading to a range of additional pathologies that extend beyond diabetes. For instance, infections with CBV can result in diverse health complications such as gastrointestinal disorders, myocarditis, pneumonia, aseptic meningitis, encephalitis, and hepatitis (69). Similarly, EMCV can also cause myocarditis and mural and valvular endocarditis and RCMV can also cause viral retinitis, myocarditis, and endocarditis (54, 70). In addition, there are also ethical considerations to be mindful of when using viruses to investigate diabetic outcomes as viruses can spread to other laboratory animals or even the researchers themselves (33, 34, 45, 47). Each of these factors highlights the need for rigorous standards and precautions in the use of viral models for diabetes research.

### 2.4 Surgical T1D animal models

Surgical interventions have also been employed both to create animal models of T1D and to assess therapeutic strategies. Key surgical methodologies utilized in the study of T1DM include complete removal of the pancreas, (pancreatectomy), transplantation of insulin-producing islets, and surgical removal of the thymus gland (thymectomy) (34, 71). These animal models are described below and summarized in **Table 4**.

To study the consequences of pancreatic β cell loss, researchers have performed complete pancreatectomy in both small and large animals to assess the detrimental effects of insulin deficiency on glucose metabolism and the eventual development of diabetes in both small and large animals (22, 49, 71) (**Table 4**). The Islet transplantation surgical approach involves the implantation of clusters of insulin-producing pancreatic β cells into other organs, such as liver, in the recipient animals. Upon glucose stimulation, these transplanted islets secrete insulin, contributing to the restoration of glucose homeostasis. This surgical approach has been shown to be useful for investigating immune responses to islet transplantation and the therapeutic assessment of survival of transplanted islets in small and large T1DM animals (22, 71) (**Table 4**). Thymectomy surgical models have been invaluable for researchers investigating the autoreactive T lymphocytes that mediate the autoimmune destruction of pancreatic β cells in T1D (34, 71). The importance of these models lies in the thymus’s key role in T cell development and the process by which T cells become educated to recognize self from non-self antigens.

These surgical animal models for T1D research come with a set of limitations, such as a significant loss of animals due to surgical complications, the necessity for highly skilled surgeons to perform the operations, and considerable cost, among other limitations (22, 49).

## 3 Type 2 diabetic mellitus animal models

The main type 2 diabetic mellitus (T2DM) animal models include chemically induced T2DM animal models, genetically/spontaneously induced T2DM animal models, surgically induced T2DM animal models, and diet/nutrition induced T2DM animal models. These models are discussed in the following sections and summarized in **Tables 5**–**8**.

**Table 5:** Chemically induced T2D animal models.

Animal	Chemical	Advantages	Limitations
**Rat**	Alloxan at sub-diabetogenic doses STZ* at sub-diabetogenic doses STZ in combination with nicotinamide	Cost-effective (23) Easy to handle and house (23) Shares several pathophysiology and pathological features with T2D in human (23) A reduced amount of stress by human contact (23)	Similar limitations in murine models as discussed below. The susceptibility to alloxan and STZ varies among different species and even among different strains of the same species. This can limit the generalizability of the findings (72). Off-target effects (25, 73). STZ and alloxan typically induce an acute model of diabetes, which does not represent the chronic nature of T2DM in humans (74). Different anatomic features, such as loose skin with dense hair in murine skins (23, 24). Different physiological features. For example, wound healing occurs primarily via wound contraction in murine models as opposed to reepithelization in human skin (23, 24) Hyperglycemia mainly develops through the direct cytotoxic action on pancreatic β-cells and insulin deficiency rather than the adverse effects of insulin resistance (24, 26)
**Mouse**	Alloxan at sub-diabetogenic doses STZ* at sub-diabetogenic doses STZ in combination with nicotinamide	Cost-effective (23) Easy to house (23) Shares numerous pathophysiology and pathological features with humans (23) Less rate of morbidity (23)	
**Rabbit**	Alloxan STZ STZ plus nicotinamide	More cost effective than other larger animals (23, 24) Similar metabolic and pathological profile of burn wound in humans (23, 24)	Difficult to produce irreversible diabetes (27) Risk of infections and morbidity compared to mouse and rat (23, 24)
**Pig**	STZ STZ plus nicotinamide	Anatomy and physiology of diabetes matches closely to that of humans (27) Useful for studying T1DM* and T2DM* (27)	Expensive animal model with more cost for care and housing (23) Administration of anesthesia requires a skilled veterinarian (23) Requires higher doses to produce diabetic conditions (27) Difficult to produce irreversible diabetes (27)

*STZ, Streptozotocin; T1DM, Type 1 Diabetes Mellitus; T2DM, Type 2 Diabetes Mellitus.

**Table 6:** Genetically/spontaneously induced T2D animal models.

Animal	Description	Advantages	Limitations
db/db* mouse	Autosomal recessive point mutation, Gly-to-Thr	Most widely used mouse model in T2D research (75–80) Show hyperglycemia, hyperphagia, and insulin resistance alongside the obesity (75)	Wounds in db/db mice heal primarily by contraction, as opposed re-epithelialization seen in humans, although this limitation can be overcome using splinted wound models (81, 82). db/db mice are sterile, the maintenance of db/db mice requires breeding between heterozygous (db/+) pairs, thus adding to the cost and effort (83, 84).
Ob/ob* mouse	*ob/ob* mutant, a nonsense mutation	Used in obesity-induced T2D research (34) Used to assess drugs to counter hyperphagia associated with obesity (85)	The limitations with the ob/ob mouse model are similar to db/db mice (34, 81, 82). ob/ob males can occasionally reproduce if maintained on a restricted diet. Ob/ob females are always sterile, but their sterility is corrected via leptin treatment (86).
GK* rat	Repeated inbreeding of glucose intolerant Wistar rats (87)	Best used to study T2DM due to their liver and skeletal muscle insulin resistance (88) Finding treatments to advance β cell function (27) Finding treatments to advance β cell survival (27)	Early β cell destruction remains a limitation for mimicking T2DM (26)
OSHR* rat	Hypertensive female rats mated with normotensive male rats over multiple generations (85)	Used to study the relationship between endocrine and metabolic abnormalities to obesity (85) Useful for examining the relationship of arthrosclerosis with high blood pressure and hyperlipemia (89)	Rats sometimes forced to consume a high-calorie diet to become diabetic (90)
**Akita mouse**	Ins-2 autosomal dominant mutation	Investigate albuminuria and glomerular mesangial expansion (41) Studying potential alleviators of chronic stress in pancreatic islet; therefore, making some useful for investigating T2DM* (41) Model can also be used to study T1DM (41)	Not confirmed by anti-diabetic or anti-neuropathic drugs (91) Underlying mechanism for the increase of mesangial matrix is currently unknown, and therefore, observations for mesangial immunoglobulin A deposits is of limited value (92)
**Zucker fatty rat**	Mating of the Sherman and Merck stock M rats; thus, inherit an autosomal recessive mutation	Most widely used rat model for T2D (85) Utilized as a model of human obesity accompanied with hyperlipidemia and hypertension (24, 26)	Develops severe diabetes in only males (24, 93)
**Zucker diabetic fatty rat**	Missense mutation in the gene coding the leptin receptor (fa/fa)	Studying the development from prediabetic to diabetic state in T2DM (94) Shows functional and morphological renal lesions that closely resemble the ones seen in humans with T2DM (95)	Complete descriptions of insulin resistance are unknown (96)
ZDSD* rat	Developed by crossing two different rat models and selectively bred for obesity and diabetes traits (97)	Important for investigating diabetic ulcer conditions (98) Used to study T2DM (97, 98)	May display an impaired renal function (98) May display progressive albuminuria (98)
**NONcNZO10 mouse**	A recombinant congenic strain that was developed at The Jackson Laboratory	Good model to study the etiology of obesity induced T2D and metabolic syndrome (99–101) Good model for wound healing impairment studies if splinted (99)	Only male mice develop hyperglycemia (100),
**TALLYHO/Jng (TH) mouse**	Inbred polygenic model for type 2 diabetes (T2D) with moderate obesity (102)	Exhibit many similarities with NONcNZO10/LtJ with respect to T2D phenotypes (102, 103)	Glucose intolerance and hyperglycemia are exhibited only in males, which is also a limitation of this model (102, 103).
**KK mouse**	Polygenic diabetic model characterized by moderate obesity, polyphagia, and polyuria features (104)	Represent the human condition more closely than monogenic species (24) Used to study T2DM (27, 71, 105) Used to discover insulin resistance treatment (27, 71, 105)	High cost (71, 105) Limited availability (71, 105) Possible genetic variations between individual mice (71, 105)

*db/db, diabetic/diabetic; Leptin receptor mutant homozygous; GK, Goto Kakizaki; ob/ob, obese/obese; Leptin mutant homozygous; OSHR, Obese Spontaneously Hypersensitive Rat; T1DM, Type 1 Diabetes Mellitus; T2DM, Type 2 Diabetes Mellitus; ZDSD, Zucker Diabetic-Sprague Dawley.

**Table 7:** Surgical T2D animal models .

Animal	Surgery Type	Advantage	Limitations
**Rat**	Partial pancreatomy Bariatric surgery and Renal Denervation	Reflects the isolated effects of a reduced β cell mass (48, 106, 107) High reproductive rate (23, 48, 106, 107) Cost effective, easy handling, and housing (23, 48, 106, 107)	Partial-thickness wounds are difficult to create because skin is too thin (23, 50, 106, 107) High possibly of exocrine acinar cells being excised too (48, 106, 107)
**Mouse**	Partial pancreatomy Bariatric surgery and Renal Denervation	Reflects the isolated effects of a reduced β cell mass (48) High reproductive rate (23, 48, 106, 107) Cost effective and housing (23, 48, 106, 107)	Partial-thickness wounds are difficult to create because skin is too thin (23, 50, 106, 107) High possibly of exocrine acinar cells being excised too (48, 106, 107)
**Zebrafish**	Partial pancreatomy Bariatric surgery and Renal Denervation	Study specific genes and similarity in genetic pathways involved in glucose metabolism (108) High reproductive capacity and rapid development (106–108) Cost effective and easy to maintain (106–108)	Live in an artificial environment (108) Have a standardized diet (49) (49, 108) High technical skills needed (49, 108)
**Dog**	Partial pancreatomy Bariatric surgery and Renal Denervation	Model is a reliable method to induce hyperglycemia (27) Large size and longer lifespan (27) Natural development of T2DM*-like conditions (27, 106, 107)	Invasive surgery (27) Increased chance of hypoglycemia (27) High cost (49) High technical skills needed (49)
**Pig**	Partial pancreatomy Bariatric surgery and Renal Denervation	Pancreatectomy followed by autotransplantation of the isolated islets largely matches that seen in human diabetics (27) Useful for studying T1DM* and T2DM (27)	Invasive surgery (23, 27) Increased chance of hypoglycemia (23, 27) High cost (49) High technical skills needed (49)
**Primate**	Partial pancreatomy Bariatric surgery and Renal Denervation	Higher lifespans (48, 52) Remarkable similarities to the human physiology (52)	Limited availability (52) Ethical concerns (48) High cost for handling and housing (52) High technical skills needed (49)

*T1DM, Type 1 Diabetes Mellitus; T2DM, Type 2 Diabetes Mellitus.

**Table 8:** Diet/nutrition induced T2D animal models.

Animal	Description	Advantage	Limitations
**Sand Rat**	Most widely used diet-induced, polygenic diabetic rat model (109)	Toxicity of chemicals on other organs can be avoided (24, 26) Utilized for studying the interaction between obesity and diabetes (24) Useful for studying the effects of diet and exercise and for pharmacological research (24)	Requiring extended periods of time for treatment (24) Not suitable for screening antidiabetic agents (24)
**Spiny Mouse**	Characteristically have massive hyperplasia of pancreatic islets and increased pancreatic insulin content (56)	Toxicity of chemicals on other organs can be avoided (24, 26) Useful for studying T2DM and its pathogenesis (24, 56)	Requiring extended periods of time for treatment (24) Not suitable for screening antidiabetic agents (24)
**C57BL/6J Mouse**	Characterized by marked obesity, hyperinsulinemia, insulin resistance and glucose intolerance (24)	Toxicity of chemicals on other organs can be avoided (24, 26) Useful for studying T2DM and its pathogenesis (24, 56)	Requiring extended periods of time for treatment (24) Not suitable for screening antidiabetic agents (24)
**Rhesus** macaque **(Monkey)**	Rapidly lose their previously accumulated adipose tissue and become ketotic; therefore, requiring insulin to survive (24) The final secretion loss is linked with deposition of amylin or amyloid in pancreatic β-cells (24)	Capable of developing characteristics of metabolic syndrome and coronary artery disease (26) Diabetic complications similar to human T2DM (24)	Requiring extended periods of time for treatment (24) Not suitable for screening antidiabetic agents (24) Limited availability (52) Ethical concerns (48) High cost for handling and housing (52)
**Gottingen Minipigs**	Require a high fat diet background to become firstly obese and afterwards to become characteristically T2DM (26)	Useful for studying T2DM and its pathogenesis (24, 56) Capable of developing characteristics of metabolic syndrome and coronary artery disease (26)	Requiring extended periods of time for treatment (24) Not suitable for screening antidiabetic agents (24)

*T2DM, Type 2 Diabetes Mellitus.

### 3.1 Chemically induced T2DM animal models

While alloxan and streptozotocin (STZ) are most commonly used to induce T1DM in laboratory animals, these agents are also used in developing T2DM animal models under certain experimental conditions. Sub-diabetogenic doses of STZ or alloxan can lead to a state of impaired glucose tolerance, which mirrors the initial phase of T2DM (110) (**Table 7**). In addition, when combined with high-fat diets (HFD), nicotinamide, or used within genetically susceptible animals, these agents induce conditions that more accurately reflect T2DM, characterized by insulin resistance and progressive β cell dysfunctions (26, 53, 55, 74, 110, 111). These combinations enable researchers to generate a more controlled and reproducible T2DM phenotype in animals. For example, the addition of nicotinamide protects pancreatic β cells from the cytotoxicity of alloxan or STZ, while still allowing the manifestation of key T2DM features such as impaired insulin secretion, β cell fatigue, and a decline in β cell mass (112–114).

The use of these chemicals in T2DM research might seem counterintuitive since T2DM is typically characterized by insulin resistance followed by a relative, rather than absolute, deficiency in insulin secretion. However, such models are valuable for dissecting the mechanisms underlying β cell failure and the onset of insulin deficiency as T2DM progresses.

Like all animal models, those induced chemically by alloxan and STZ have their own inherent limitations. Notably, the inducing agents may produce off-target effects, interfering with other biological functions and thus complicating the interpretation of outcomes (25, 73) (**Table 5**). Reproducing the human form of T2DM, which is chronic and progresses over time, is yet another challenge for chemically induced T2DM animal models. This complexity often confounds the extrapolation of findings to human T2DM conditions, particularly considering the distinct metabolic dysfunctions in humans as compared to animals (22, 26).

### 3.2 Genetically/spontaneously induced T2D animal models

There are many widely used T2DM animal models generated genetically or by spontaneous mutations. These T2DM animal models include the db/db and ob/ob obese mice, the Goto-Kakizaki (GK) rat, the Obese Spontaneously Hypertensive Rat (OSHR), the Zucker Diabetic Fatty (ZDF) rat, the NONcNZO10 mouse, the TALLYHO/Jng (TH) mouse, and the KK mice. These animal models are discussed in more details below and summarized in **Table 6**.

The db/db mouse model is the most widely used animal model in T2D research. It was created through an autosomal recessive homozygous point mutation in the leptin receptor (LepR) gene (34, 75). db/db mice are obese due to hyperphagia (excessive eating), and display insulin resistance, polydipsia (excessive thirst), and polyuria (excessive urination), and are used for obesity-induced T2DM research (75). db/db mice specifically exhibit certain aspects of human T2D that are not present in the high fat diet (HFD)-induced obese T2DM mice (described below), despite similar body mass. They exhibit adipose tissue inflammation and distinct alterations in metabolic flexibility seen in humans with T2DM (115). The db/db mice have been instrumental in exploring the pathogenesis of common human T2DM complications, such as, susceptibility to infection and wound healing impairment (76–79, 116–118). However, a limitation of db/db mouse model is its inability to fully reproduce the diabetic conditions in humans (34, 81). For example, the db/db mice primarily heal their wounds through contraction, which is in contrast with the re-epithelialization that is seen in humans, although this limitation can be overcome using splinted wound models (81, 82, 99). Another limitation is that homozygous db/db mice are sterile and the maintenance of db/db mice requires breeding between heterozygous (db/+) pairs, thus adding to the cost and effort (83, 84).

The ob/ob mouse was caused by a homozygous point mutation of cytosine to thymine in Exon 2 of the Leptin gene, which is responsible for the production of the leptin peptide hormone (119, 120). This nonsense mutation leads to the synthesis of a truncated leptin which is apparently degraded in the adipose tissue (119, 120). By approximately 4-weeks of age, the ob/ob mice become obese due to hyperphagia (26, 119, 121). These obese hyperglycemic mice are very similar to db/db in that they present with hyperglycemia, insulin resistance and glycosuria (glucose in the urine) without any ketonuria or coma, mimicking obese type 2 diabetic human condition (34, 122). In these mice, the islets of Langerhans are hypertrophied, which leads to an increase in insulin content and a general state of insulin resistance (34, 122). The limitations with the ob/ob mouse model are like db/db mice (34, 81, 82). It is worth noting that although ob/ob males can occasionally reproduce if maintained on a restricted diet, ob/ob females are always sterile, but their sterility can be corrected through repeated treatment with leptin (86).

The GK rat model was generated through repeated inbreeding of glucose intolerant Wistar rats (87). These rats are non-obese, insulin-resistant, and exhibit decreased pancreatic β cell mass (123, 124). Because the GK rats display liver and skeletal muscle insulin resistance, they are best used to study T2DM condition observed in nonobese diabetic humans (88). However, the inadequate β cell proliferation in early life limits this model to precisely represent the type 2 diabetic mellitus condition seen in humans (124).

The OSHR rat was generated from a mutation that arose after several breeding between spontaneously hypertensive Kyoto-Wistar strain female rats and normotensive Sprague-Dawley male rats (85). These rats are often used for investigating the connections between metabolic and endocrine abnormalities in obesity and for examining the links between arthrosclerosis with high blood pressure and hyperlipidemia (89). A limitation of this model is that overfeeding on a high-calorie diet is required for these rats to display diabetic conditions (90).

The Zucker fatty rat was originally developed in the 1960s following the discovery of a spontaneous missense mutation in the recessive fa/fa genotype (125). This mutation occurred in the leptin receptor gene and rendered it nonfunctional (85). This rat develops the same pathophysiological characteristics of obese T2DM, displayed by db/db mice, including insulin resistance and hypoinsulinemia, hypertension, hyperlipidemia, and obesity (24, 94, 95, 126). It is largely utilized as a model of human obesity accompanied with hyperlipidemia and hypertension (24, 26). The Zucker fatty rat model offers specific advantages for type 2 diabetes (T2D) research primarily due to its spontaneous nature, closely mimicking common human T2DM characteristics such as hyperglycemia, obesity, hyperphagia, polyuria, insulin disorders, and dyslipidemia (127). Additionally, these rats are also noted for being calm and easy to handle, which facilitates their use in various experimental settings (127, 128). However, there are also limitations to the Zucker fatty rat model. One significant limitation is that these animals exhibit only mild glucose intolerance which makes their utility as a T2D model questionable, given that human T2D typically involves more severe glucose intolerance (128). Furthermore, while the lipid profile is altered in these rats, they show an increase in very low-density lipoproteins (VLDL) and high-density lipoproteins (HDL), but not in low-density lipoprotein (LDL) cholesterol (127, 128). Therefore, they are not suitable models for atherogenesis studies. Additionally, despite having high systolic blood pressure values after 28 weeks, they are not considered a model for hypertension, which can be a significant comorbidity in human T2D (127, 128). It is worth noting that selective inbreeding of fa/fa rat for hyperglycemia resulted in the development of the ZDF rat model, which develops more severe diabetes than the Zucker fatty rats but only in male mice, thus reducing some of these limitations (24, 93). The ZDF rat also displays functional and morphological renal lesions that closely resemble the ones seen in humans with T2DM (95).

The Zucker Diabetic-Sprague Dawley rat developed by crossing two different rat models and selectively bred for obesity and diabetes traits (97). This model is an inbred polygenic model for obesity, diabetes, and diabetic complications (97). The ZDSD presents with hyperglycemia, hyperlipemia, hypertension, and insulin resistance features that are also displayed in human diabetics; thus, making this rat important for investigating diabetic ulcer conditions. This obese-diabetic rat model expresses T2DM in the presence of an intact leptin pathway (98). The ZDSD rats can display an impaired renal function and progressive albuminuria (98).

This approach of using genetically altered models to study diabetes has some major disadvantages. Some animal sets may take a long time to develop diabetes and display symptoms (95). Not knowing when an animal will develop diabetes can impact experimental design and data collection (34). There are also ethical considerations to note when trying to genetically manipulate and selectively breed animals (95). Developing and maintaining genetically induced animal sets can require specialized breeding environments (27, 98).

The NONcNZO10 mouse is a recombinant congenic strain that was developed at The Jackson Laboratory by combining six well-known diabesity quantitative trait loci (QTL) from New Zealand Obese (NZO/HlLt) strain with the Nonobese Nondiabetic (NON/LtJ) mice. The male mice develop type 2 diabetes characterized by maturity onset obesity, hyperglycemia, and insulin resistance (100). This strain was specifically developed to serve as a model for the etiology of T2D and for studying human obesity induced T2D and metabolic syndrome (99–101). Type 2 diabetes in males of this strain results from polygenic interactions producing a moderate obesity rather than the massive obesity elicited by mutations in the leptin or leptin receptor axis, such as ob/ob or db/db mice. Unlike mice with monogenic obesity syndromes, NONcNZO10/LtJ, males do not display hypercortisolism, hyperphagia, or obvious thermoregulatory defects (129). When male mice from this strain are weaned onto a chow diet containing 10-11% fat by weight, they develop visceral obesity, maturity-onset hyperglycemia, dyslipidemia, moderate liver steatosis, and pancreatic islet atrophy (99–101). This mouse is quite useful as a model to study the etiology of obesity induced T2D and metabolic syndrome (99–101). In addition, NONcNZO10/LtJ appears to be a superior model for wound healing impairment studies, as it closely resembles the defects in wound healing that is characteristic of diabetic patients (99). There are also limitations for the use of this animal model. In addition to its high cost, only male mice develop hyperglycemia, thereby restricting the applicability of this model in gender-related studies concerning Type 2 diabetes (100).

The TALLYHO/Jng (TH) mouse is an inbred polygenic model for type 2 diabetes with moderate obesity (102, 130). They exhibit many similarities with NONcNZO10/LtJ with respect to T2DM phenotypes but there are also differences between these mice (102, 130). Both male and female TH mice are characterized by increased body and fat pad weights, hyperleptinemia, hyperinsulinemia, and hyperlipidemia, but glucose intolerance and hyperglycemia are exhibited only in males, which is also a limitation of this model (102, 103).

The KK mice are a polygenic model for non-insulin-dependent diabetes mellitus (NIDDM). They develop hereditary diabetes with a progression from a prediabetic stage to full diabetes, including renal, retinal, and neurological complications (131). These mice exhibit hyperglycemia, glucose intolerance, and microalbuminuria (71). Both genetic predisposition and environmental factors influence these phenotypes. In the prediabetic stage, KK mice develop mild to moderate glomerulosclerosis which progresses to severe glomerulosclerosis and proteinuria later in life (131). These characteristics can be transmitted to normal control mice through back-crossing, indicating that a specific genetic trait(s) is essential for the development of diabetes. Consistent with this notion, a prior investigation a quantitative trait locus (QTL) for fasting glucose levels on chromosome 6 and three loci on chromosomes 3, 5, and 14 associated with glucose tolerance and plasma insulin levels in these mice (104). The KK mice have been useful in discovering novel treatments to improve insulin resistance (27). Some drawbacks for using this mouse model to study diabetes include its high expense, limited availability, and its possible genetic variations between individual mice due to its non-homogenous genetic nature (71, 105).

### 3.3 Surgical T2D animal models

Surgical techniques have also been used to generate animal models for T2DM. These techniques generally aim to reduce pancreatic β cell mass (22, 71). The main T2DM surgical models include partial pancreatectomy models, bariatric surgery models, and the renal denervation models (22, 71, 106, 107). The surgical T2DM animal models have been invaluable in shedding lights on many aspects of T2DM in human. However, their invasive nature and the potential to harm healthy tissue and the requirement for highly skilled investigators, are major limitations, reducing their utility in research (34). These animal models are discussed in more details below and summarized in **Table 7**.

Partial pancreatectomy was a technique established several decades ago by researchers who wished to induce diabetes in animals while avoiding liver and kidney damages that can be caused by administration of alloxan or streptozotocin (132, 133). Over time, many modifications have been made to this procedure. These include variations in the percentage of pancreas removal and the integration of partial pancreatectomy with chemical induction methods (54, 134, 135). This model has been useful for investigating the implications of the loss of pancreatic β cells and for assessing the regenerative capability of β cells or their progenitors which are relevant to T2D conditions in human (34, 54, 134, 135).

Renal denervation surgery involves the disruption of the nerves in the renal artery (136). This procedure is primarily used to treat resistant hypertension, but it has also been used to study its potential therapeutic effects on cardiovascular, metabolic, and renal functions in diabetic animals (137–139). The renal denervation model assists researchers to explore the part that sympathetic nervous system plays in T2DM development (138).

Bariatric surgery describes various surgical procedures that are used to reduce obesity and manage obesity-related conditions (140). This can be accomplished by reduction of stomach size, reduction of nutrient absorption, or a combination of both (140, 141). Bariatric surgeries do not cause diabetes, rather, they are used in diabetic animals by investigators to study the effects of weight reduction on improving insulin sensitivity and glucose metabolism (54, 142, 143).

These surgical models also have limitations that should be considered prior to use. One major limitation is their invasive nature and their potential to cause damage to healthy organs, resulting in pain and stress to animals (34). The triggered inflammatory and immune reactions associated with these surgical interventions may also confound the results of the research (107). Another limitation is the requirement for highly skilled investigators to perform such surgeries, precisely to avoid causing additional harm to animals. Also, there can be a variability in outcomes as results may vary based on the years of experience a researcher may have with performing surgical experiments (22, 107). Finally, ethical concerns related to animal suffering and wellbeing should also be considered when deciding whether or not to employ such techniques (34, 71).

### 3.4 Diet/nutrition induced T2D animal models

Obesity greatly increases the likelihood of developing prediabetes and T2DM, especially when linked with a higher concentration of fat in the abdominal and intra-abdominal areas, as well as elevated triglyceride levels in the liver and muscles, because it can lead to both insulin resistance and β cell dysfunction (144, 145). Naturally, investigators have employed several diet and nutritional strategies to generate T2DM animal models to study T2DM disease progression and for testing potential treatments.

One common approach is using a high-fat diet (HFD) to induce obesity and insulin resistance in rodents (146–150). This method attempts to mimic the Western diet, which typically contains significantly more saturated fat than recommended dietary guidelines (151). Like the diabetes seen in humans, animals in this category tend to develop diabetes linked to obesity because of overnutrition (24, 56). The primary animal models used in this group include the sand rat, spiny mouse, C57/BL 6J mouse, Rhesus macaque monkey and the Gottingen minipig (24, 26, 56). The non-rodent models, Rhesus macaque monkeys and Gottingen minipigs, are unique because, when fed with an atherogenic diet, they can develop characteristics of metabolic syndrome and coronary artery disease (26).

The HFD is routinely combined with low dose Streptozotocin (STZ) to generate HFD/STZ animal models of T2DM (152). This involves feeding animals a HFD to induce hyperinsulinemia and insulin resistance, followed by an injection of a low dose of STZ to slowly reduce pancreatic β cell mass, leading to a decrease in insulin production and mimicking the pathophysiology associated with T2DM in human (152). The HFD/STZ is particularly a good animal model for the later stage of the disease, depending on the amount of residual β cell mass (153).

Another nutritional method that is frequently used to induce T2DM involves feeding mice energy-dense (ED) diets, such as higher intake of processed red meat and sugary desserts and drinks (154, 155). This strategy has been shown in various animals to lead to glucose intolerance, hyperglycemia, increased glycosylated hemoglobin (HbA1c), and glycosuria, typically within a 30-week period (154–156).

There are also several drawbacks to this approach (24, 26). One major limitation is the extended time required for the diet/nutrition treatment to induce diabetes (24). Additionally, this method may not induce hyperglycemia in genetically normal animals, rendering it less effective for testing antidiabetic agents that target blood glucose levels (24). The complexity of human diets can also be challenging to replicate in diet/nutrition-induced models, potentially requiring long-term studies to observe chronic effects and gather reliable data (56). Another major drawback is controlling all variables like individual metabolic behavior, which can confound the results of the experiment (24, 56).

### 3.5 Concluding remarks and future prospective

As the incidence of diabetes increases globally, the need for continued research for more effective treatment options is critical. Type 1 (T1DM) and Type 2 diabetes mellitus (T2DM) are the predominant forms of diabetes, accounting for most cases worldwide. These two types, while sharing some common features, also exhibit distinct pathophysiological mechanisms, thus necessitating targeted research approaches for each. Animal models have provided important insights into the genetic, environmental, and immunological factors that contribute to the development and progression of diabetes. Given their fundamental differences, investigating T1DM and T2DM requires tailored experimental designs, using animal models that closely mimic the specific diabetes type. Therefore, the selection of an appropriate animal model is critical in understanding the disease process and in testing potential therapeutic interventions.

Although animal models have played an essential role in advancing diabetes research, it is important to note that currently, there are no animal models capable of fully replicating human type 1 and type 2 diabetic conditions, and they all come with inherent limitations that necessitate careful consideration. This underscores the critical need for ongoing research efforts to improve these animal models. This understanding is crucial for developing more effective and tailored treatments, ultimately aiming to improve the quality of life for individuals living with diabetes. Moreover, ethical considerations are paramount in animal research. Researchers must adhere to strict guidelines to ensure ethical treatment of animals, including minimizing discomfort and using alternative approaches whenever possible to reduce animal use in research.

## Author contributions

RS: Writing – original draft, Writing – review & editing. MG: Writing – review & editing. SS: Conceptualization, Funding acquisition, Investigation, Supervision, Writing – review & editing, Writing – original draft.

## References

[1] Ong KL Stafford LK McLaughlin SA Boyko EJ Vollset SE Smith AE. Global, regional, and national burden of diabetes from 1990 to 2021, with projections of prevalence to 2050: a systematic analysis for the Global Burden of Disease Study 2021. Lancet. (2023) 402(10397):203–34. doi: 10.1016/S0140-6736(23)01301-6 PMC10364581 37356446

[2] Ozougwu J Obimba K Belonwu C Unakalamba C. The pathogenesis and pathophysiology of type 1 and type 2 diabetes mellitus. J Physiol Pathophysiol. (2013) 4:46–57. doi: 10.5897/JPAP.

[3] l. R P Sagen JV Bjørkhaug L Odili S Shehadeh N Bakry D. Molven: Permanent neonatal diabetes caused by glucokinase deficiency: inborn error of the glucose-insulin signaling pathway. Diabetes. (2003) 52:2854–60. doi: 10.2337/diabetes.52.11.2854. 14578306

[4] Gregg EW Sattar N Ali MK. The changing face of diabetes complications. Lancet Diabetes Endocrinol. (2016) 4:537–47. doi: 10.1016/S2213-8587(16)30010-9. 27156051

[5] Harding JL Pavkov ME Magliano DJ Shaw JE Gregg EW. Global trends in diabetes complications: a review of current evidence. Diabetologia. (2019) 62:3–16. doi: 10.1007/s00125-018-4711-2. 30171279

[6] Vigneri P Frasca F Sciacca L Pandini G Vigneri R. Diabetes and cancer. Endocrine-related Cancer. (2009) 16:1103–23. doi: 10.1677/ERC-09-0087. 19620249

[7] Ducat L Philipson LH Anderson BJ. The mental health comorbidities of diabetes. Jama. (2014) 312:691–2. doi: 10.1001/jama.2014.8040. PMC4439400 25010529

[8] Nagendra L Boro H Mannar V. Bacterial infections in diabetes. Endotext [Internet]. (2022).

[9] Lao M Li C Li J Chen D Ding M Gong Y. Opportunistic invasive fungal disease in patients with type 2 diabetes mellitus from Southern China: clinical features and associated factors. J Diabetes Invest. (2020) 11:731–44. doi: 10.1111/jdi.13183. PMC7232281 31758642

[10] Rodrigues CF Rodrigues ME Henriques M. Candida sp. infections in patients with diabetes mellitus. J Clin Med. (2019) 8:76. doi: 10.3390/jcm8010076. 30634716 PMC6352194

[11] Klekotka RB Mizgała E Król W. The etiology of lower respiratory tract infections in people with diabetes. Adv Respir Med. (2015) 83:401–8. doi: 10.5603/PiAP.2015.0065. 26379004

[12] Op de Beeck A Eizirik DL. Viral infections in type 1 diabetes mellitus—why the β cells? Nat Rev Endocrinol. (2016) 12:263–73. doi: 10.1038/nrendo.2016.30. PMC5348720 27020257

[13] Zhang X Zhu X Ji Y Li H Hou F Xiao C. Increased risk of hepatitis B virus infection amongst individuals with diabetes mellitus. Bioscience Reports. (2019) 39(3):BSR20181715. doi: 10.1042/BSR20181715. 30858308 PMC6438870

[14] Mobasseri M Shirmohammadi M Amiri T Vahed N Fard HH Ghojazadeh M. Prevalence and incidence of type 1 diabetes in the world: a systematic review and meta-analysis. Health promotion Perspect. (2020) 10:98. doi: 10.34172/hpp.2020.18. PMC7146037 32296622

[15] Skyler JS Bakris GL Bonifacio E Darsow T Eckel RH Groop L. Differentiation of diabetes by pathophysiology, natural history, and prognosis. Diabetes. (2017) 66:241–55. doi: 10.2337/db16-0806. PMC5384660 27980006

[16] W. H. Organization. Classification of diabetes mellitus. Geneva, Switzerland: World Health Organization (WHO) (2019).

[17] Yuan S Larsson SC. An atlas on risk factors for type 2 diabetes: a wide-angled Mendelian randomisation study. Diabetologia. (2020) 63:2359–71. doi: 10.1007/s00125-020-05253-x. PMC7527357 32895727

[18] Kyrou I Tsigos C Mavrogianni C Cardon G Van Stappen V Latomme J. Sociodemographic and lifestyle-related risk factors for identifying vulnerable groups for type 2 diabetes: a narrative review with emphasis on data from Europe. BMC endocrine Disord. (2020) 20:1–13. doi: 10.1186/s12902-019-0463-3. PMC7066728 32164656

[19] Kelly J Karlsen M Steinke G. Type 2 diabetes remission and lifestyle medicine: a position statement from the American College of Lifestyle Medicine. Am J lifestyle Med. (2020) 14:406–19. doi: 10.1177/1559827620930962. PMC7692017 33281521

[20] Garcia-Molina L Lewis-Mikhael A-M Riquelme-Gallego B Cano-Ibanez N Oliveras-Lopez M-J Bueno-Cavanillas A. Improving type 2 diabetes mellitus glycaemic control through lifestyle modification implementing diet intervention: a systematic review and meta-analysis. Eur J Nutr. (2020) 59:1313–28. doi: 10.1007/s00394-019-02147-6. 31781857

[21] Kleinert M Clemmensen C Hofmann SM Moore MC Renner S Woods SC. Animal models of obesity and diabetes mellitus. Nat Rev Endocrinol. (2018) 14:140–62. doi: 10.1038/nrendo.2017.161. 29348476

[22] King A Bowe J. Animal models for diabetes: understanding the pathogenesis and finding new treatments. Biochem Pharmacol. (2016) 99:1–10. doi: 10.1016/j.bcp.2015.08.108. 26432954

[23] Sanapalli BKR Yele V Singh MK Krishnamurthy PT Karri VVSR. Preclinical models of diabetic wound healing: A critical review. Biomedicine Pharmacotherapy. (2021) 142:111946. doi: 10.1016/j.biopha.2021.111946. 34339915

[24] Srinivasan K Ramarao P. Animal models in type 2 diabetes research: an overview. Indian J Med Res. (2007) 125:451–72. 17496368

[25] Lee JH Yang SH Oh JM Lee MG. Pharmacokinetics of drugs in rats with diabetes mellitus induced by alloxan or streptozocin: comparison with those in patients with type I diabetes mellitus. J Pharm Pharmacol. (2010) 62:1–23. doi: 10.1211/jpp.62.01.0001. 20722995

[26] Chatzigeorgiou A Halapas A Kalafatakis K Kamper E. The use of animal models in the study of diabetes mellitus. In Vivo. (2009) 23:245–58. 19414410

[27] King AJ. The use of animal models in diabetes research. Br J Pharmacol. (2012) 166:877–94. doi: 10.1111/j.1476-5381.2012.01911.x PMC3417415 22352879

[28] Pearson JA Wong FS Wen L. The importance of the Non Obese Diabetic (NOD) mouse model in autoimmune diabetes. J Autoimmun. (2016) 66:76–88. doi: 10.1016/j.jaut.2015.08.019. 26403950 PMC4765310

[29] Von Herrath MG Nepom GT. Lost in translation: barriers to implementing clinical immunotherapeutics for autoimmunity. In: Rockefeller Univ Press. (2005) 202(9):1159–62. doi: 10.1084/jem.20051224. PMC2213225 16275758

[30] Yokoi N Namae M Fuse M Wang H-Y Hirata T Seino S. Establishment and characterization of the Komeda diabetes-prone rat as a segregating inbred strain. Exp Anim. (2003) 52:295–301. doi: 10.1538/expanim.52.295. 14562605

[31] Yokoi N Hayashi C Fujiwara Y Wang H-Y Seino S. Genetic reconstitution of autoimmune type 1 diabetes with two major susceptibility genes in the rat. Diabetes. (2007) 56:506–12. doi: 10.2337/db06-1027. 17259398

[32] Kawano K Hirashima T Mori S Saitoh Y Kurosumi M Natori T. New inbred strain of Long-Evans Tokushima lean rats with IDDM without lymphopenia. Diabetes. (1991) 40:1375–81. doi: 10.2337/diabetes.40.11.1375. 1682194

[33] Mordes JP Bortell R Blankenhorn EP Rossini AA Greiner DL. Rat models of type 1 diabetes: genetics, environment, and autoimmunity. ILAR J. (2004) 45:278–91. doi: 10.1093/ilar.45.3.278. 15229375

[34] Kottaisamy CPD Raj DS Prasanth Kumar V Sankaran U. Experimental animal models for diabetes and its related complications—a review. Lab Anim Res. (2021) 37:1–14. doi: 10.1186/s42826-021-00101-4. 34429169 PMC8385906

[35] Weiss H Bleich A Hedrich H-J Kölsch B Elsner M Jörns A. Genetic analysis of the LEW. 1AR1-iddm rat: an animal model for spontaneous diabetes mellitus. Mamm Genome. (2005) 16:432–41. doi: 10.1007/s00335-004-3022-8. 16075370

[36] Lenzen S Tiedge M Elsner M Lortz S Weiss H Jörns A. The LEW. 1AR1/Ztm-iddm rat: a new model of spontaneous insulin-dependent diabetes mellitus. Diabetologia. (2001) 44:1189–96. doi: 10.1007/s001250100625. 11596676

[37] Pandey S Dvorakova MC. Future perspective of diabetic animal models. Endocrine Metab Immune Disord Drug Targets. (2020) 20:25. doi: 10.2174/1871530319666190626143832. PMC7360914 31241444

[38] Crisá L Mordes JP Rossini AA. Autoimmune diabetes mellitus in the BB rat. Diabetes/metabolism Rev. (1992) 8:9–37. doi: 10.1002/dmr.5610080104. 1633738

[39] Yoshioka M Kayo T Ikeda T Koizuni A. A novel locus, Mody4, distal to D7Mit189 on chromosome 7 determines early-onset NIDDM in nonobese C57BL/6 (Akita) mutant mice. Diabetes. (1997) 46:887–94. doi: 10.2337/diab.46.5.887. 9133560

[40] Wang J Takeuchi T Tanaka S Kubo S-K Kayo T Lu D. A mutation in the insulin 2 gene induces diabetes with severe pancreatic β-cell dysfunction in the Mody mouse. J Clin Invest. (1999) 103:27–37. doi: 10.1172/JCI4431. 9884331 PMC407861

[41] Gurley SB Clare SE Snow KP Hu A Meyer TW Coffman TM. Impact of genetic background on nephropathy in diabetic mice. Am J Physiology-Renal Physiol. (2006) 290(1):214–22. doi: 10.1152/ajprenal.00204.2005. 16118394

[42] Izumi T Yokota-Hashimoto H Zhao S Wang J Halban PA Takeuchi T. Dominant negative pathogenesis by mutant proinsulin in the Akita diabetic mouse. Diabetes. (2003) 52:409–16. doi: 10.2337/diabetes.52.2.409. 12540615

[43] Nir T Melton DA Dor Y. Recovery from diabetes in mice by β cell regeneration. J Clin Invest. (2007) 117:2553–61. doi: 10.1172/JCI32959. PMC1957545 17786244

[44] Horwitz MS Bradley LM Harbertson J Krahl T Lee J Sarvennick N. Diabetes induced by Coxsackie virus: initiation by bystander damage and not molecular mimicry. Nat Med. (1998) 4:781–5. doi: 10.1038/nm0798-781. 9662368

[45] Jaeckel E Manns M Von Herrath M. Viruses and diabetes. Ann New York Acad Sci. (2002) 958:7–25. doi: 10.1111/j.1749-6632.2002.tb02943.x. 12021080

[46] Filippi CM von Herrath MG. Viral trigger for type 1 diabetes: pros and cons. Diabetes. (2008) 57:2863. doi: 10.2337/db07-1023. 18971433 PMC2570378

[47] Yoon J-W Austin M Onodera T Notkins AL. Virus-induced diabetes mellitus: isolation of a virus from the pancreas of a child with diabetic ketoacidosis. New Engl J Med. (1979) 300:1173–9. doi: 10.1056/NEJM197905243002102. 219345

[48] Thisted L Østergaard MV Pedersen AA Pedersen PJ Lindsay RT Murray AJ. Rat pancreatectomy combined with isoprenaline or uninephrectomy as models of diabetic cardiomyopathy or nephropathy. Sci Rep. (2020) 10:1–15. doi: 10.1038/s41598-020-73046-8. 32999377 PMC7527487

[49] Etuk E. Animals models for studying diabetes mellitus. Agric Biol JN Am. (2010) 1:130–4.

[50] Davidson J. Animal models for wound repair. Arch Dermatol Res. (1998) 290:S1–S11. doi: 10.1007/PL00007448. 9710378

[51] Calasans-Maia MD Monteiro ML Áscoli FO Granjeiro JM. The rabbit as an animal model for experimental surgery. Acta cirurgica Bras. (2009) 24:325–8. doi: 10.1590/S0102-86502009000400014. 19705034

[52] Zakaria ZZ Ahmad MN Qinna NA. Animal models in type 2 diabetes mellitus research: pros and cons. Jordan J Agric Sci. (2021) 17:425–40. doi: 10.35516/jjas.v17i4.

[53] Federiuk IF Casey HM Quinn MJ Wood MD Ward KW. Induction of type-1 diabetes mellitus in laboratory rats by use of alloxan: route of administration, pitfalls, and insulin treatment. Comp Med. (2004) 54:252–7. 15253270

[54] Junod A Lambert A Orci L Pictet R Gonet A Renold A. Studies of the diabetogenic action of streptozotocin. Proc Soc Exp Biol Med. (1967) 126:201–5. doi: 10.3181/00379727-126-32401. 4864021

[55] Eleazu CO Eleazu KC Chukwuma S Essien UN. Review of the mechanism of cell death resulting from streptozotocin challenge in experimental animals, its practical use and potential risk to humans. J Diabetes Metab Disord. (2013) 12:1–7. doi: 10.1186/2251-6581-12-60. 24364898 PMC7962474

[56] Kumar S Singh R Vasudeva N Sharma S. Acute and chronic animal models for the evaluation of anti-diabetic agents. Cardiovasc Diabetol. (2012) 11:1–13. doi: 10.1186/1475-2840-11-9. 22257465 PMC3286385

[57] Kroin JS Buvanendran A Li J Moric M Im H-J Tuman KJ. Short-term glycemic control is effective in reducing surgical site infection in diabetic rats. Anesth Analgesia. (2015) 120:1289–96. doi: 10.1213/ANE.0000000000000650. 25695673

[58] Kroin JS Li J Goldufsky JW Gupta KH Moghtaderi M Buvanendran A. Perioperative high inspired oxygen fraction therapy reduces surgical site infection with. Pseudomonas aeruginosa rats. J Med Microbiol. (2016) 65:738–44. doi: 10.1099/jmm.0.000295. PMC5756492 27302326

[59] Kouhkheil R Fridoni M Piryaei A Taheri S Chirani AS Anarkooli IJ. The effect of combined pulsed wave low-level laser therapy and mesenchymal stem cell-conditioned medium on the healing of an infected wound with methicillin-resistant Staphylococcal aureus in diabetic rats. J Cell Biochem. (2018) 119:5788–97. doi: 10.1002/jcb.26759 29574990

[60] Kroin JS Li J Shafikhani S Gupta KH Moric M Buvanendran A. Local vancomycin effectively reduces surgical site infection at implant site in rodents. Regional Anesth Pain Med. (2018) 43:795–804. doi: 10.1097/AAP.0000000000000820. 29905629

[61] Soleimani H Amini A Taheri S Sajadi E Shafikhani S Schuger LA. The effect of combined photobiomodulation and curcumin on skin wound healing in type I diabetes in rats. J Photochem Photobiol B: Biol. (2018) 181:23–30. doi: 10.1016/j.jphotobiol.2018.02.023. 29486459

[62] Ahmadi H Bayat M Gazor R Asadi R Gachkar L Rezaei F. Impact of preconditioned diabetic stem cells and photobiomodulation on quantity and degranulation of mast cells in a delayed healing wound simulation in type one diabetic rats. Lasers Med Sci. (2021 37(3):P1593–1604. doi: 10.1007/s10103-021-03408-9 34476655

[63] Szkudelski T. The mechanism of alloxan and streptozotocin action in B cells of the rat pancreas. Physiol Res. (2001) 50:537–46. 11829314

[64] Rohilla A Ali S. Alloxan induced diabetes: mechanisms and effects. Int J Res Pharm Biomed Sci. (2012) 3:819–23.

[65] Bacevic M Rompen E Radermecker R Drion P Lambert F. Practical considerations for reducing mortality rates in alloxan-induced diabetic rabbits. Heliyon 6(6). (2020). doi: 10.1016/j.heliyon.2020.e04103. PMC7305394 32577551

[66] Weiss H Arndt T Jörns A Lenzen S Cuppen E Hedrich HJ. The mutation of the LEW. 1AR1-iddm rat maps to the telomeric end of rat chromosome 1. Mamm Genome. (2008) 19:292–7. doi: 10.1007/s00335-008-9102-4. 18357488

[67] Nekoua MP Alidjinou EK Hober D. Persistent coxsackievirus B infection and pathogenesis of type 1 diabetes mellitus. Nat Rev Endocrinol. (2022) 18:503–16. doi: 10.1038/s41574-022-00688-1. PMC9157043 35650334

[68] Craighead JE McLane MF. Diabetes mellitus: induction in mice by encephalomyocarditis virus. Science. (1968) 162:913–4. doi: 10.1126/science.162.3856.913. 4300932

[69] Tariq N Kyriakopoulos C. Group B coxsackie virus. (2020). 32809618

[70] Carocci M Bakkali-Kassimi L. The encephalomyocarditis virus. Virulence. (2012) 3:351–67. doi: 10.4161/viru.20573. PMC3478238 22722247

[71] Chen C Cohrs CM Stertmann J Bozsak R Speier S. Human beta cell mass and function in diabetes: Recent advances in knowledge and technologies to understand disease pathogenesis. Mol Metab. (2017) 6:943–57. doi: 10.1016/j.molmet.2017.06.019. PMC5605733 28951820

[72] Rossini AA Like AA Chick WL Appel MC Cahill G Jr. Studies of streptozotocin-induced insulitis and diabetes. Proc Natl Acad Sci. (1977) 74:2485–9. doi: 10.1073/pnas.74.6.2485. PMC432197 142253

[73] Like AA Rossini AA. Streptozotocin-induced pancreatic insulitis: new model of diabetes mellitus. Science. (1976) 193:415–7. doi: 10.1126/science.180605. 180605

[74] Reed M Meszaros K Entes L Claypool M Pinkett J Gadbois T. A new rat model of type 2 diabetes: the fat-fed, streptozotocin-treated rat. Metabolism-Clinical Exp. (2000) 49:1390–4. doi: 10.1053/meta.2000.17721. 11092499

[75] Rai V Moellmer R Agrawal DK. Clinically relevant experimental rodent models of diabetic foot ulcer. Mol Cell Biochem. (2022) 477:1239–47. doi: 10.1007/s11010-022-04372-w. 35089527

[76] Roy R Zayas J Singh SK Delgado K Wood SJ Mohamed MF. Overriding impaired FPR chemotaxis signaling in diabetic neutrophil stimulates infection control in murine diabetic wound. Elife. (2022) 11:1–25. doi: 10.7554/eLife.72071 PMC8846594 35112667

[77] Roy R Zayas J Mohamed MF Aboonabi A Delgado K Wallace J. IL-10 dysregulation underlies chemokine insufficiency, delayed macrophage response, and impaired healing in diabetic wounds. J Invest Dermatol. (2022) 142:692–704.e14. doi: 10.1016/j.jid.2021.08.428 34517005 PMC8860852

[78] Goldufsky J Wood SJ Jayaraman V Majdobeh O Chen L Qin S. *Pseudomonas aeruginosa* uses T3SS to inhibit diabetic wound healing. Wound Repair Regener. (2015) 23:557–64. doi: 10.1111/wrr.12310 PMC4690211 25912785

[79] Roy R Mahmud F Zayas J Kuzel T Reiser J Shafikhani S. Reduced bioactive microbial products (PAMPs) contribute to dysregulated immune responses and impaired healing in infected wounds in diabetic mice. J Invest Dermatol. (2023) S0022-202X:02512. doi: 10.1016/j.jid.2023.08.004 PMC10840742 37619833

[80] Guest PC Rahmoune H. Characterization of the db/db mouse model of type 2 diabetes. Pre-Clinical Models: Techniques Protoc. (2019), 195–201. doi: 10.1007/978-1-4939-8994-2. 30535696

[81] Suriano F Vieira-Silva S Falony G Roumain M Paquot A Pelicaen R. Novel insights into the genetically obese (ob/ob) and diabetic (db/db) mice: Two sides of the same coin. Microbiome. (2021) 9:1–20. doi: 10.1186/s40168-021-01097-8. 34183063 PMC8240277

[82] Goodson WH III Hunt TK. Deficient collagen formation by obese mice in a standard wound model. Am J Surg. (1979) 138:692–4. doi: 10.1016/0002-9610(79)90350-7. 495858

[83] Zhang Y Hu M Ma H Qu J Wang Y Hou L. The impairment of reproduction in db/db mice is not mediated by intraovarian defective leptin signaling. Fertility sterility. (2012) 97:1183–91. doi: 10.1016/j.fertnstert.2012.01.126. 22341640

[84] Peng B-y Wang Q Luo Y-h He J-f Tan T Zhu H. : A novel and quick PCR-based method to genotype mice with a leptin receptor mutation (db/db mice). Acta Pharmacologica Sin. (2018) 39:117–23. doi: 10.1038/aps.2017.52. PMC5758667 28748911

[85] Lakshmanan M Shewade DG Raj GM. Introduction to basics of pharmacology and toxicology: volume 3: experimental pharmacology: research methodology and biostatistics. Springer. (2022). doi: 10.1007/978-981-19-5343-9.

[86] Chehab FF Lim ME Lu R. Correction of the sterility defect in homozygous obese female mice by treatment with the human recombinant leptin. Nat Genet. (1996) 12:318–20. doi: 10.1038/ng0396-318. 8589726

[87] GoTo Y Kakizaki M Masaki N. Spontaneous diabetes produced by selective breeding of normal Wistar rats. Proc Japan Acad. (1975) 51:80–5. doi: 10.2183/pjab1945.51.80.

[88] Chen H Charlat O Tartaglia LA Woolf EA Weng X Ellis SJ. Evidence that the diabetes gene encodes the leptin receptor: identification of a mutation in the leptin receptor gene in db/db mice. Cell. (1996) 84:491–5. doi: 10.1016/S0092-8674(00)81294-5. 8608603

[89] Koletsky S. Obese spontaneously hypertensive rats—a model for study of atherosclerosis. Exp Mol Pathol. (1973) 19:53–60. doi: 10.1016/0014-4800(73)90040-3. 4721724

[90] Miesel A Müller H Thermann M Heidbreder M Dominiak P Raasch W. Overfeeding-induced obesity in spontaneously hypertensive rats: an animal model of the human metabolic syndrome. Ann Nutr Metab. (2010) 56:127–42. doi: 10.1159/000278748. 20134158

[91] Islam M. Animal models of diabetic neuropathy: progress since 1960s. J Diabetes research. (2013) 2013:P149452. doi: 10.1155/2013/149452. PMC3745837 23984428

[92] Haseyama T Fujita T Hirasawa F Tsukada M Wakui H Komatsuda A. Complications of IgA nephropathy in a non-insulin-dependent diabetes model, the Akita mouse. Tohoku J Exp Med. (2002) 198:233–44. doi: 10.1620/tjem.198.233. 12630555

[93] Garnett KE Chapman P Chambers JA Waddell ID Boam DS. Differential gene expression between Zucker Fatty rats and Zucker Diabetic Fatty rats: a potential role for the immediate-early gene Egr-1 in regulation of beta cell proliferation. J Mol Endocrinol. (2005) 35:13–26. doi: 10.1677/jme.1.01792. 16087718

[94] Wexler B Iams S McMurtry J. Pathophysiological differences between obese and non-obese spontaneously hypertensive rats. Br J Exp Pathol. (1980) 61:195. 7426376 PMC2041508

[95] Kong L-l Wu H Cui W-p Zhou W-h Luo P Sun J. Advances in murine models of diabetic nephropathy. J Diabetes research. (2013) 2013. doi: 10.1155/2013/797548. PMC3697778 23844375

[96] Leonard B Watson R Loomes K Phillips A Cooper G. Insulin resistance in the Zucker diabetic fatty rat: a metabolic characterisation of obese and lean phenotypes. Acta diabetologica. (2005) 42:162. doi: 10.1007/s00592-005-0197-8. 16382303

[97] Han L Bittner S Dong D Cortez Y Bittner A Chan J. Molecular changes in hepatic metabolism in ZDSD rats–A new polygenic rodent model of obesity, metabolic syndrome, and diabetes. Biochim Biophys Acta (BBA)-Molecular Basis Dis. (2020) 1866:165688. doi: 10.1016/j.bbadis.2020.165688. PMC8229451 31987840

[98] Peterson RG Van Jackson C Zimmerman KM. The ZDSD rat: a novel model of diabetic nephropathy. Am J Trans Res. (2017) 9:4236. doi: 10.1111/j.1524-475X.2010.00634.x PMC5622266 28979697

[99] Fang RC Kryger ZB Buck DW de la Garza 2M Galiano RD Mustoe TA. Limitations of the db/db mouse in translational wound healing research: Is the NONcNZO10 polygenic mouse model superior? Wound Repair Regener. (2010) 18:605–13. doi: 10.1111/wrr.2010.18.issue-6 20955341

[100] Cho Y-R Kim H-J Park S-Y Ko HJ Hong E-G Higashimori T. Hyperglycemia, maturity-onset obesity, and insulin resistance in NONcNZO10/LtJ males, a new mouse model of type 2 diabetes. Am J Physiology-Endocrinology Metab. (2007) 293:E327–36. doi: 10.1152/ajpendo.00376.2006. 17616608

[101] Halaas JL Boozer C Blair-West J Fidahusein N Denton DA Friedman JM. Physiological response to long-term peripheral and central leptin infusion in lean and obese mice. Proc Natl Acad Sci. (1997) 94:8878–83. doi: 10.1073/pnas.94.16.8878. PMC23177 9238071

[102] Kim JH Saxton AM. The TALLYHO mouse as a model of human type 2 diabetes. Anim Models Diabetes Research. (2012) P75–87. doi: 10.1007/978-1-62703-068-7_6 22893402

[103] Leiter EH Strobel M O'Neill A Schultz D SChile A Reifsnyder PC. Comparison of two new mouse models of polygenic type 2 diabetes at the Jackson Laboratory, NONcNZO10Lt/J and TALLYHO/JngJ. J Diabetes research. (2013) 2013:P1–7. doi: 10.1155/2013/165327. PMC3647594 23671854

[104] Suto J-i Matsuura S Imamura K Yamanaka H Sekikawa K. Genetic analysis of non-insulin-dependent diabetes mellitus in KK and KK-Ay mice. Eur J Endocrinol. (1998) 139:654–61. doi: 10.1530/eje.0.1390654. 9916873

[105] Iizuka Y Kim H Nakasatomi M Matsumoto A Shimizu J. Phenotypic and genotypic changes in obesity and type 2 diabetes of male KK mice with aging. Exp Anim. (2022) 71:71–81. doi: 10.1538/expanim.21-0109. 34588391 PMC8828408

[106] Wang Y Rijal B Xu M Li Z An Y Zhang F. Renal denervation improves vascular endothelial dysfunction by inducing autophagy via AMPK/mTOR signaling activation in a rat model of type 2 diabetes mellitus with insulin resistance. Acta Diabetologica. (2020) 57:1227–43. doi: 10.1007/s00592-020-01532-6. 32488498

[107] Mistry SB Omana JJ Kini S. Rat models for bariatric surgery and surgery for type 2 diabetes mellitus. Obes Surg. (2009) 19:655–60. doi: 10.1007/s11695-009-9811-0. 19266247

[108] Zang L Shimada Y Nishimura N. Development of a novel zebrafish model for type 2 diabetes mellitus. Sci Rep. (2017) 7:1461. doi: 10.1038/s41598-017-01432-w. 28469250 PMC5431185

[109] Ziv E Shafrir E Kalman R Galer S Bar-On H. Changing pattern of prevalence of insulin resistance in Psammomys obesus, a model of nutritionally induced type 2 diabetes. Metabolism. (1999) 48:1549–54. doi: 10.1016/S0026-0495(99)90244-5. 10599987

[110] Wang Z Gleichmann H. GLUT2 in pancreatic islets: crucial target molecule in diabetes induced with multiple low doses of streptozotocin in mice. Diabetes. (1998) 47:50–6. doi: 10.2337/diabetes.47.1.50. 9421374

[111] Masiello P Broca C Gross R Roye M Manteghetti M Hillaire-Buys D. Experimental NIDDM: development of a new model in adult rats administered streptozotocin and nicotinamide. Diabetes. (1998) 47:224–9. doi: 10.2337/diabetes.47.2.224. 9519717

[112] Kahn SE. The importance of β-cell failure in the development and progression of type 2 diabetes. J Clin Endocrinol Metab. (2001) 86:4047–58. doi: 10.1210/jcem.86.9.7713. 11549624

[113] Butler AE Janson J Bonner-Weir S Ritzel R Rizza RA Butler PC. β-cell deficit and increased β-cell apoptosis in humans with type 2 diabetes. Diabetes. (2003) 52:102–10. doi: 10.2337/diabetes.52.1.102. 12502499

[114] Prentki M Nolan CJ. Islet β cell failure in type 2 diabetes. J Clin Invest. (2006) 116:1802–12. doi: 10.1172/JCI29103. PMC1483155 16823478

[115] Burke SJ Batdorf HM Burk DH Noland RC Eder AE Boulos MS. db/db mice exhibit features of human type 2 diabetes that are not present in weight-matched C57BL/6J mice fed a western diet. J Diabetes research. (2017) 2017:P1–17. doi: 10.1155/2017/8503754. PMC5606106 29038790

[116] Mohamed MF Gupta K Goldufsky JW Roy R Callaghan LT Wetzel DM. CrkII/Abl phosphorylation cascade is critical for NLRC4 inflammasome activity and is blocked by *Pseudomonas aeruginosa* ExoT. Nat Commun. (2022) 13:1–16. doi: 10.1038/s41467-022-28967-5. 35277504 PMC8917168

[117] Wood S Jayaraman V Huelsmann EJ Bonish B Burgad D Sivaramakrishnan G. Pro-inflammatory chemokine CCL2 (MCP-1) promotes healing in diabetic wounds by restoring the macrophage response. PloS One. (2014) 9:e91574. doi: 10.1371/journal.pone.0091574 24618995 PMC3950222

[118] Roy R Mahmud F Zayas J Kuzel TM Reiser J Shafikhani SH. Reduced bioactive microbial products (Pathogen-associated molecular patterns) contribute to dysregulated immune responses and impaired healing in infected wounds in mice with diabetes. J Invest Dermatol. (2024) 144:387–397.e11. doi: 10.1016/j.jid.2023.08.004 37619833 PMC10840742

[119] Zhang Y Proenca R Maffei M Barone M Leopold L Friedman JM. Positional cloning of the mouse obese gene and its human homologue. Nature. (1994) 372:425–32. doi: 10.1038/372425a0. 7984236

[120] Friedman JM Leibel R Siegel D Walsh J Bahary N. Molecular mapping of the mouse ob mutation. Genomics. (1991) 11:1054–62. doi: 10.1016/0888-7543(91)90032-A. 1686014

[121] Tomita T Doull V Pollock H Krizsan D. Pancreatic islets of obese hyperglycemic mice (ob/ob). Pancreas. (1992) 7:367–75. doi: 10.1097/00006676-199205000-00015. 1594559

[122] Halaas JL Gajiwala KS Maffei M Cohen SL Chait BT Rabinowitz D. Weight-reducing effects of the plasma protein encoded by the obese gene. Science. (1995) 269:543–6. doi: 10.1126/science.7624777. 7624777

[123] Cefalu WT. Animal models of type 2 diabetes: clinical presentation and pathophysiological relevance to the human condition. ILAR J. (2006) 47:186–98. doi: 10.1093/ilar.47.3.186. 16804194

[124] Movassat J Calderari S Fernández E Martín M Escrivá F Plachot C. Type 2 diabetes–a matter of failing β-cell neogenesis? Clues from the GK rat model. Diabetes Obes Metab. (2007) 9:187–95. doi: 10.1111/j.1463-1326.2007.00786.x. 17919193

[125] Bray GA. The Zucker-fatty rat: a review. In: Fed Proc. (1977) 36(2):P148–153. 320051

[126] Phillips MS Liu Q Hammond HA Dugan V Hey PJ Caskey CT. Leptin receptor missense mutation in the fatty Zucker rat. Nat Genet. (1996) 13:18–9. doi: 10.1038/ng0596-18. 8673096

[127] Capcarova M Kalafova A. Zucker diabetic fatty rats for research in diabetes. Anim. Model Med Biol. (2020), 1–18.

[128] Aleixandre A Miguel M. Zucker rats as an experimental model for the study of various diseases. Endocrinología y Nutrición: órgano la Sociedad Española Endocrinología y Nutrición. (2008) 55:217–22. doi: 10.1016/S1575-0922(08)70670-3. 22967915

[129] Hirata T Yoshitomi T Inoue M Iigo Y Matsumoto K Kubota K. Pathological and gene expression analysis of a polygenic diabetes model, NONcNZO10/LtJ mice. Gene. (2017) 629:52–8. doi: 10.1016/j.gene.2017.07.075. 28760554

[130] Denvir J Boskovic G Fan J Primerano DA Parkman JK Kim JH. Whole genome sequence analysis of the TALLYHO/Jng mouse. BMC Genomics. (2016) 17:1–15. doi: 10.1186/s12864-016-3245-6. 27835940 PMC5106808

[131] Reddi A Camerini-Davalos R. Hereditary diabetes in the KK mouse: an overview. Prediabetes. (1988) 246:P7–15. doi: 10.1007/978-1-4684-5616-5_2 3074669

[132] Penhos J Krahl M. Insulin stimulus of leucine incorporation in rat liver protein. Am J Physiology-Legacy Content. (1962) 202:349–52. doi: 10.1152/ajplegacy.1962.202.2.349. 14485087

[133] Kiely ML. The effect of cortisone on the rate of eruption, DNA synthesis, and mitotic activity in the maxillary incisor of the female albino rat. (1967) 1–162.

[134] Peshavaria M Larmie BL Lausier J Satish B Habibovic A Roskens V. Regulation of pancreatic β-cell regeneration in the normoglycemic 60% partial-pancreatectomy mouse. Diabetes. (2006) 55:3289–98. doi: 10.2337/db06-0017 17130472

[135] Saleh M Sharma K Kalsi R Fusco J Sehrawat A Saloman JL. Chemical pancreatectomy treats chronic pancreatitis while preserving endocrine function in preclinical models. J Clin investigation 131(3). (2021). doi: 10.1172/JCI143301. PMC7843231 33351784

[136] Singh RR Denton KM. Renal Denervation: A treatment for hypertension and chronic kidney disease. Hypertension. (2018) 72:528–36. doi: 10.1161/HYPERTENSIONAHA.118.10265. 30354761

[137] Luippold G Beilharz M Mühlbauer B. Chronic renal denervation prevents glomerular hyperfiltration in diabetic rats. Nephrol Dialysis Transplant. (2004) 19:342–7. doi: 10.1093/ndt/gfg584. 14736957

[138] Dias LD Casali KR Leguisamo NM Azambuja F Souza MS Okamoto M. Renal denervation in an animal model of diabetes and hypertension: Impact on the autonomic nervous system and nephropathy. Cardiovasc Diabetol. (2011) 10:1–8. doi: 10.1186/1475-2840-10-33. 21496329 PMC3110548

[139] de Oliveira TL Lincevicius GS Shimoura CG Simoes-Sato AY Garcia ML Bergamaschi CT. Effects of renal denervation on cardiovascular, metabolic and renal functions in streptozotocin-induced diabetic rats. Life Sci. (2021) 278:119534. doi: 10.1016/j.lfs.2021.119534. 33933461

[140] Rogers AM. Current state of bariatric surgery: procedures, data, and patient management. Techniques Vasc Interventional Radiol. (2020) 23:100654. doi: 10.1016/j.tvir.2020.100654. 32192634

[141] Pucci A Batterham R. Mechanisms underlying the weight loss effects of RYGB and SG: similar, yet different. J endocrinological Invest. (2019) 42:117–28. doi: 10.1007/s40618-018-0892-2. PMC6394763 29730732

[142] Andreelli F Amouyal C Magnan C Mithieux G. What can bariatric surgery teach us about the pathophysiology of type 2 diabetes? Diabetes Metab. (2009) 35:499–507. doi: 10.1016/S1262-3636(09)73456-1. 20152734

[143] Lutz TA Bueter M. The use of rat and mouse models in bariatric surgery experiments. Front Nutr. (2016) 3:25. doi: 10.3389/fnut.2016.00025. 27547753 PMC4974272

[144] Klein S Gastaldelli A Yki-Järvinen H Scherer PE. Why does obesity cause diabetes? Cell Metab. (2022) 34:11–20. doi: 10.1016/j.cmet.2021.12.012. 34986330 PMC8740746

[145] Lazar MA. How obesity causes diabetes: not a tall tale. Science. (2005) 307:373–5. doi: 10.1126/science.1104342. 15662001

[146] Atamni HJA-T Mott R Soller M Iraqi FA. High-fat-diet induced development of increased fasting glucose levels and impaired response to intraperitoneal glucose challenge in the collaborative cross mouse genetic reference population. BMC Genet. (2016) 17:1–19. doi: 10.1186/s12863-015-0321-x. 26728312 PMC4700737

[147] Stott NL Marino JS. High fat rodent models of type 2 diabetes: from rodent to human. Nutrients. (2020) 12:3650. doi: 10.3390/nu12123650. 33261000 PMC7761287

[148] Howell GE III Mulligan C Meek E Chambers JE. Effect of chronic p, p′-dichlorodiphenyldichloroethylene (DDE) exposure on high fat diet-induced alterations in glucose and lipid metabolism in male C57BL/6H mice. Toxicology. (2015) 328:112–22. doi: 10.1016/j.tox.2014.12.017. PMC6490679 25541407

[149] Lozano I van der Werf R Bietiger W Seyfritz E Peronet C Pinget M. High-fructose and high-fat diet-induced disorders in rats: impact on diabetes risk, hepatic and vascular complications. Nutr Metab. (2016) 13:1–13. doi: 10.1186/s12986-016-0074-1. PMC4766713 26918024

[150] Kuwabara WMT Panveloski-Costa AC Yokota CNF Pereira JNB Filho JM Torres RP. Comparison of Goto-Kakizaki rats and high fat diet-induced obese rats: Are they reliable models to study Type 2 Diabetes mellitus? PloS One. (2017) 12:e0189622. doi: 10.1371/journal.pone.0189622. 29220408 PMC5722336

[151] Clemente-Suárez VJ Beltrán-Velasco AI Redondo-Flórez L Martín-Rodríguez A Tornero-Aguilera JF. Global impacts of western diet and its effects on metabolism and health: A narrative review. Nutrients. (2023) 15:2749. doi: 10.3390/nu15122749. 37375654 PMC10302286

[152] Skovso S. Modeling type 2 diabetes in rats using high fat diet and streptozotocin. J Diabetes Investig. (2014) 5:349–58. doi: 10.1111/jdi.12235. PMC4210077 25411593

[153] Fang J-Y Lin C-H Huang T-H Chuang S-Y. *In vivo* rodent models of type 2 diabetes and their usefulness for evaluating flavonoid bioactivity. Nutrients. (2019) 11:530. doi: 10.3390/nu11030530. 30823474 PMC6470730

[154] Rizkalla SW. Glycemic index: is it a predictor of metabolic and vascular disorders? Curr Opin Clin Nutr Metab Care. (2014) 17:373–8. doi: 10.1097/MCO.0000000000000070. 24878873

[155] Riccardi G Rivellese AA Giacco R. Role of glycemic index and glycemic load in the healthy state, in prediabetes, and in diabetes. Am J Clin Nutr. (2008) 87:269S–74S. doi: 10.1093/ajcn/87.1.269S. 18175767

[156] Morris JL Bridson TL Alim MA Rush CM Rudd DM Govan BL. Development of a diet-induced murine model of diabetes featuring cardinal metabolic and pathophysiological abnormalities of type 2 diabetes. Biol Open. (2016) 5:1149–62. doi: 10.1242/bio.016790. PMC5004603 27402965

